# Supplementary material for: HSP90B1 regulates autophagy via PI3K/AKT/mTOR signaling, mediating HNSC biological behaviors
Source: PeerJ. 2024 Apr 5;12:e17028. doi: 10.7717/peerj.17028 (PMC11000640; doi:10.7717/peerj.17028)

Raw data of WB results

Figure 1. WB for detecting differential expression of HSP90B1 in HNSC cell lines.
Experimental repetition greater than or equal to 3 times

Figure 2. WB for detecting apoptosis.
Experimental repetition 3 times

Figure 4. WB for PI3K/AKT/mTOR signaling pathway.
Experimental repetition 3 times

Figure 4. WB for autophagy.
Experimental repetition 3 times

Figure 1. WB for detecting differential expression of HSP90B1 in HNSC cell lines.

Experimental repetition greater than or equal to 3 times

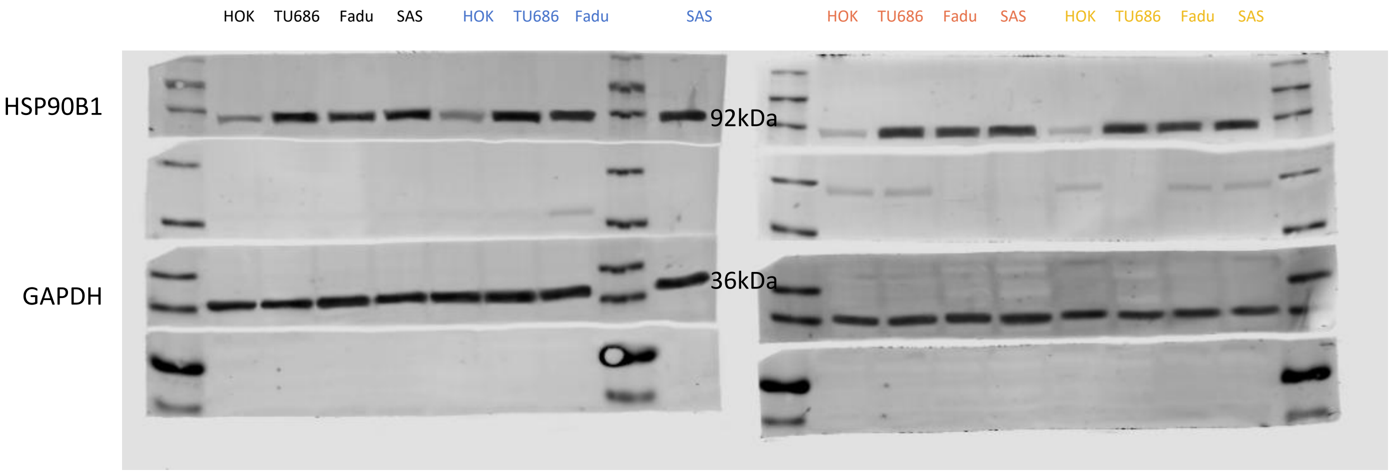

Here is the WB mark.Item No.:SW177-02

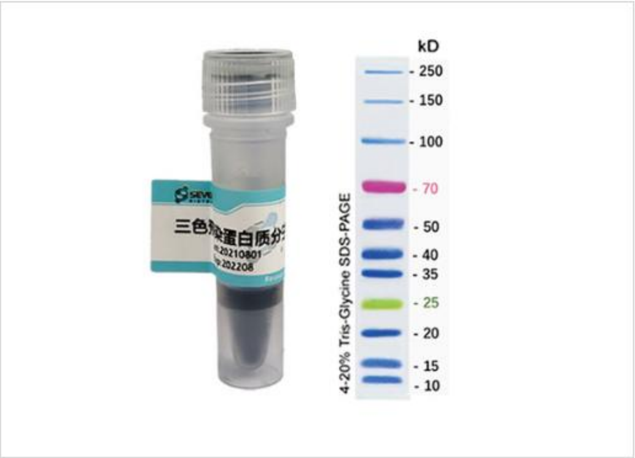

三色预染蛋白质分子量标准(10-250kDa)

品牌：Seven

价格：¥270 ~~¥380~~

货号规格：货号：SW177-02 规格：250uL

购买数量：1

加入购物车

说明书下载

Figure 2. WB for detecting apoptosis.
SiHSP90B1. Experimental repetition 3 times

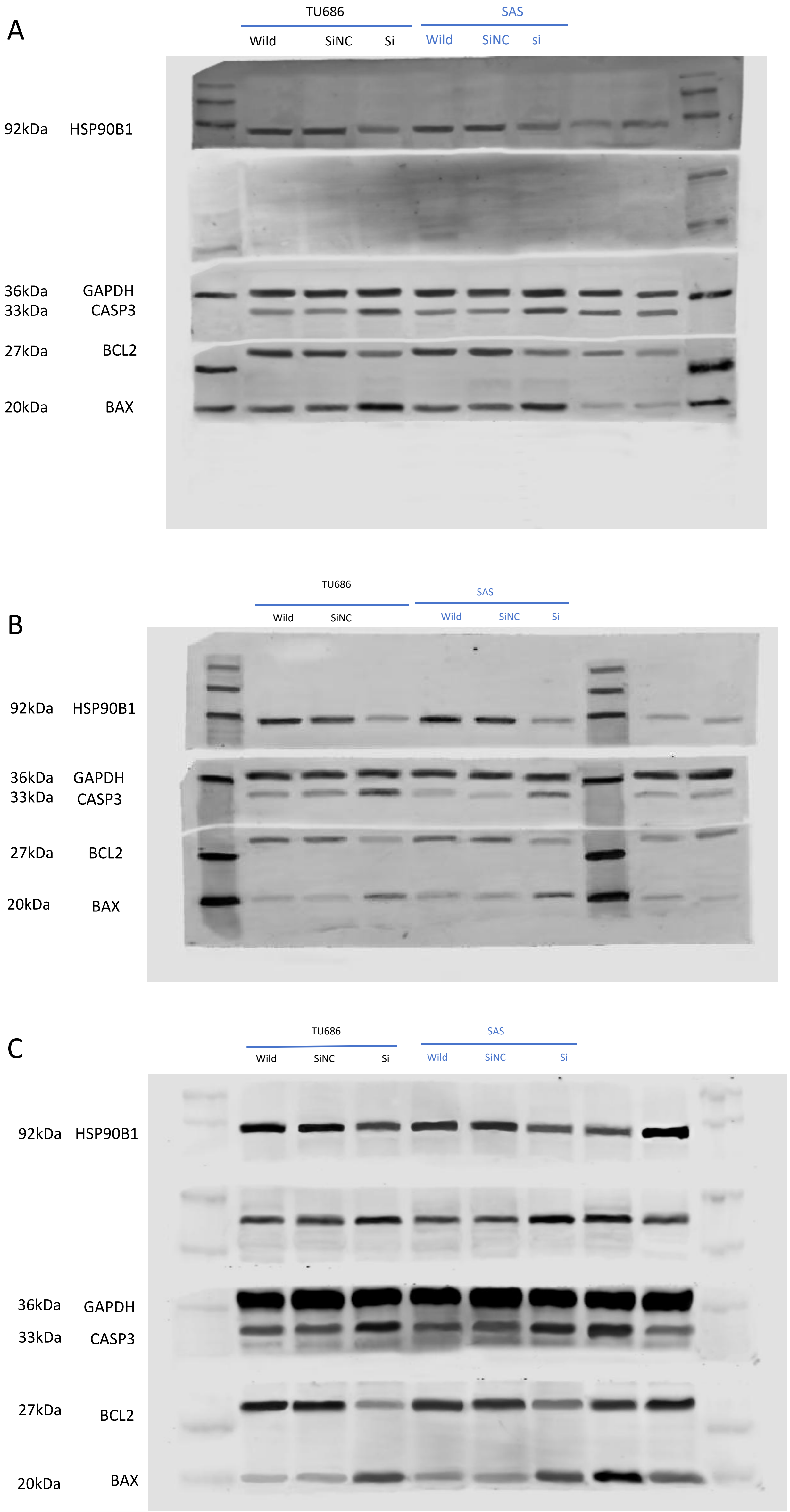

Figure 2. WB for detecting apoptosis.
OE(OverepressHSP90B1)， EV(empty vector control). Experimental repetition 3 times

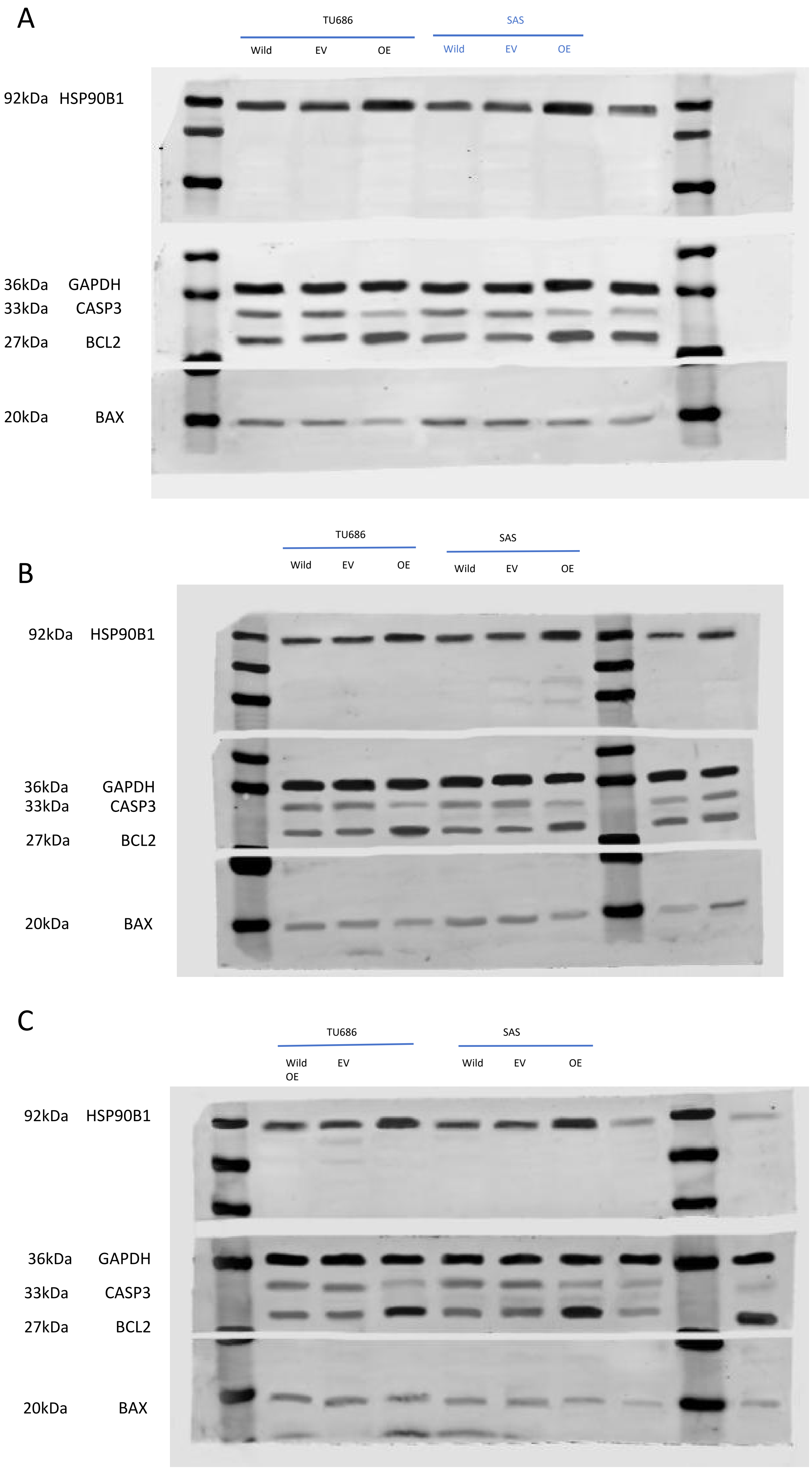

Figure 4. WB for PI3K/AKT/mTOR signaling pathway.

SiHSP90B1.Experimental repetition 3 times.

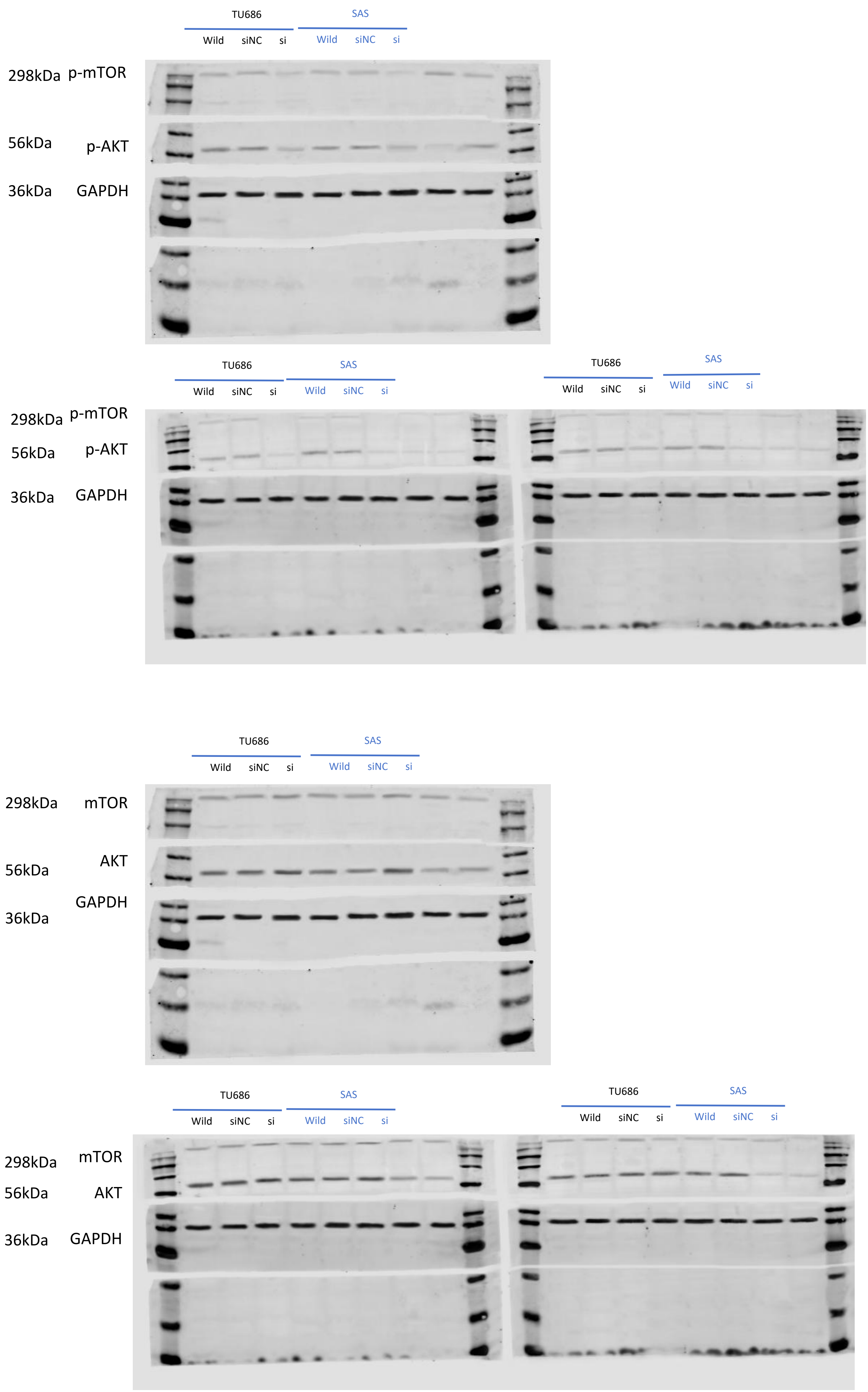

Figure 4. WB for PI3K/AKT/mTOR signaling pathway.
OverexpressHSP90B1. Experimental repetition 3 times.

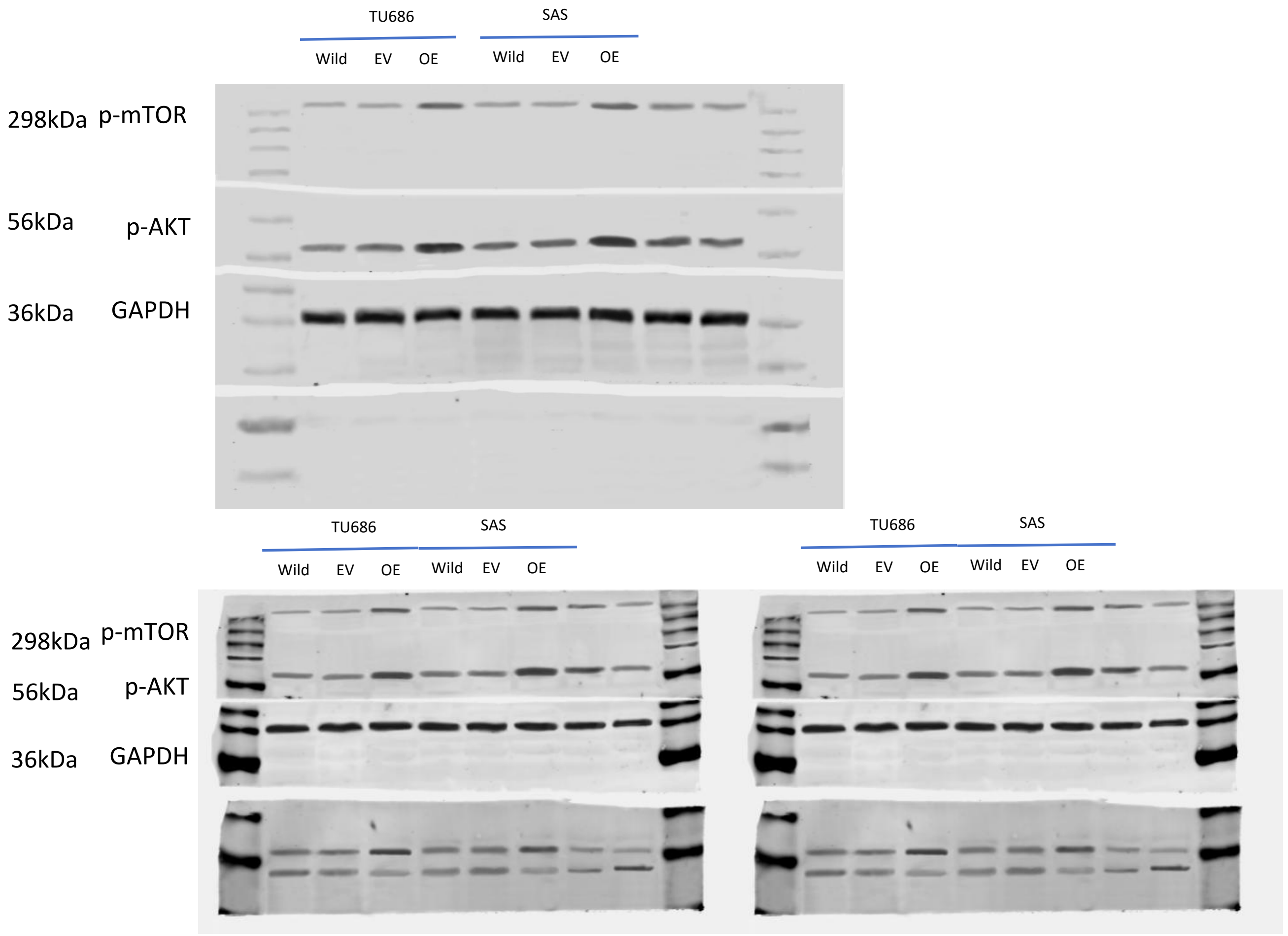

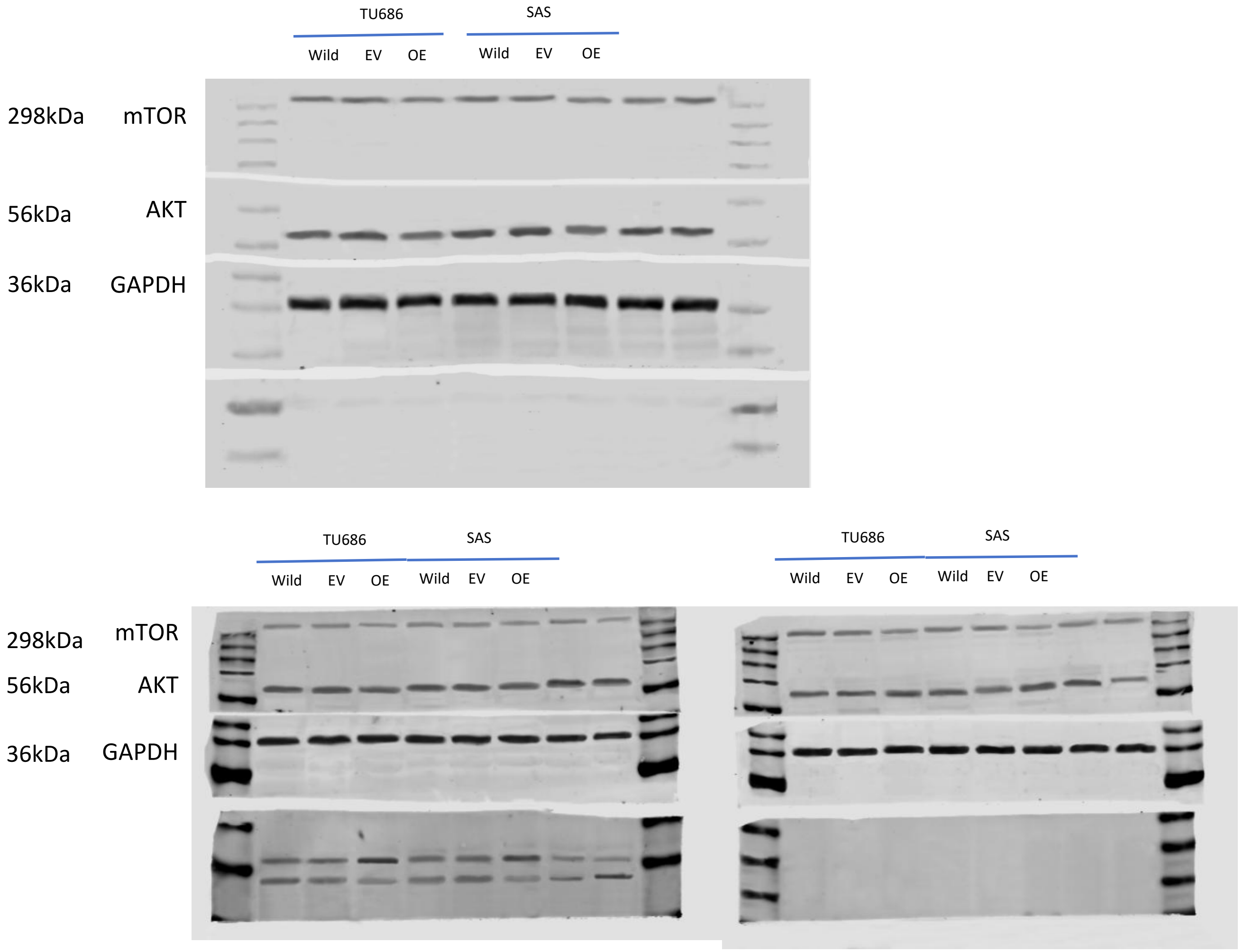

Figure 4. WB for autophagy.

SiHSP90B1. Experimental repetition 3 times

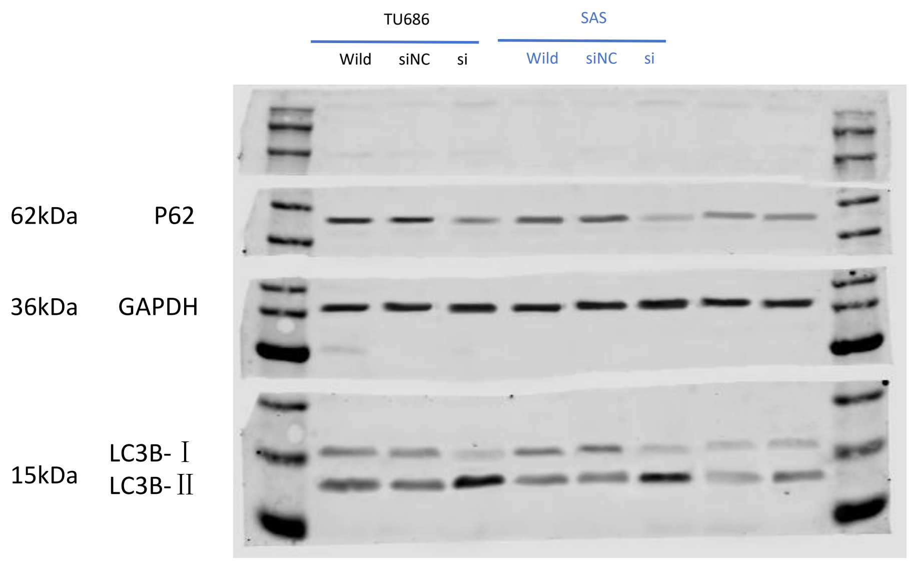

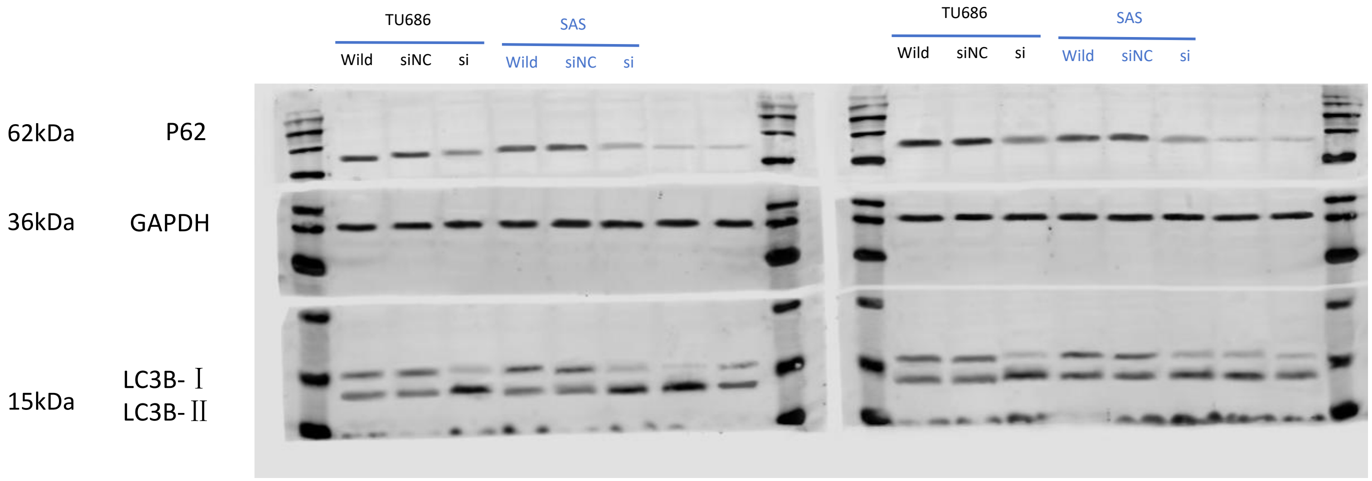

Figure 4. WB for autophagy.

OverexpressHSP90B1. Experimental repetition 3 times

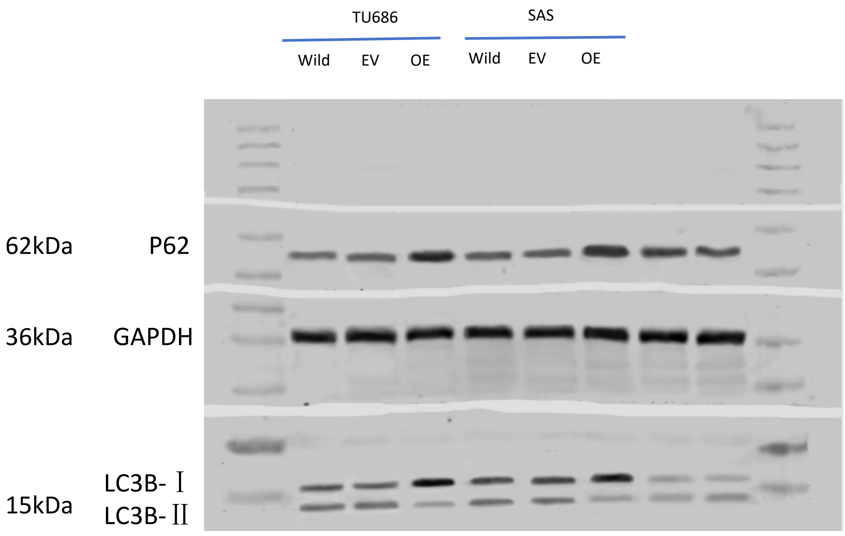

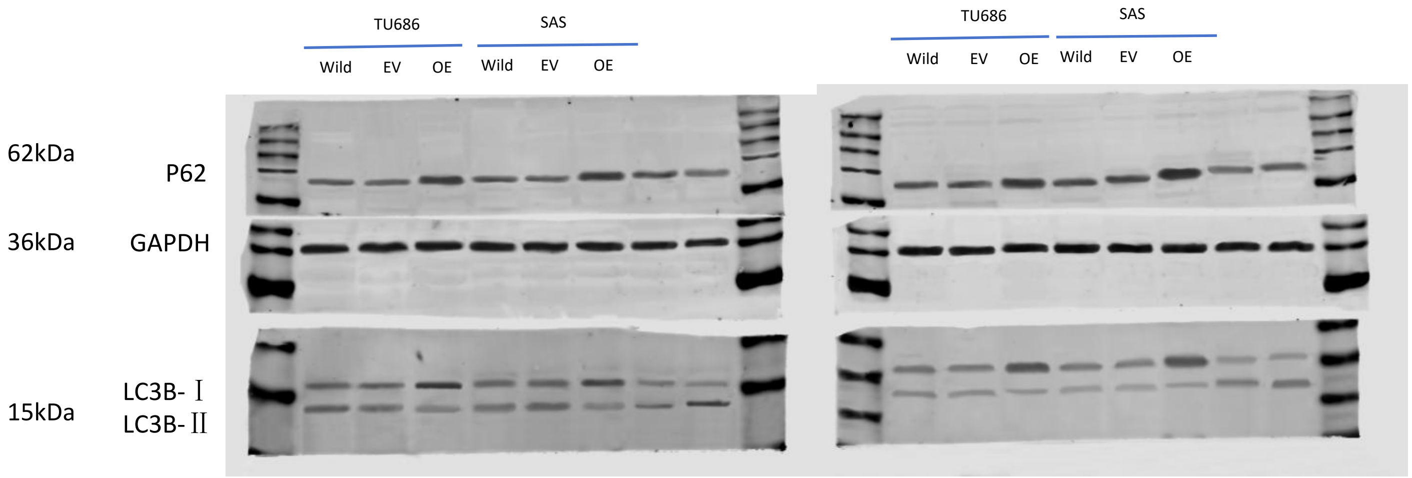

Supplement: Supplemental Information 1 — Repeated 3 times. [file peerj-12-17028-s001.zip › The raw date of WB - 上交/Raw data of WB results.pdf]
