# Supplementary figures and images for: HSP90B1 regulates autophagy via PI3K/AKT/mTOR signaling, mediating HNSC biological behaviors
Source: PeerJ. 2024 Apr 5;12:e17028. doi: 10.7717/peerj.17028 (PMC11000640; doi:10.7717/peerj.17028)

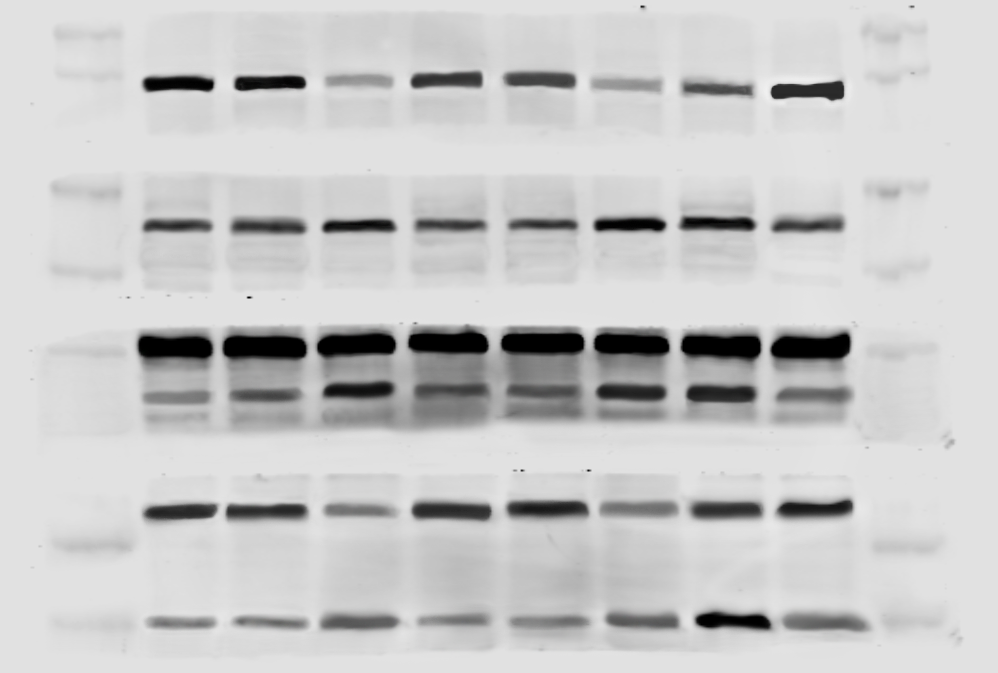

Supplement: Supplemental Information 1 — Repeated 3 times. [file peerj-12-17028-s001.zip › The raw date of WB - 上交/Figure 2 Apoptosis si 2.tif]

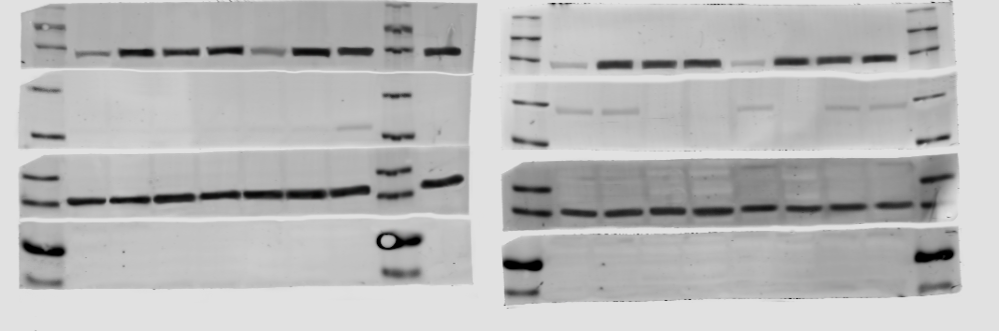

Supplement: Supplemental Information 1 — Repeated 3 times. [file peerj-12-17028-s001.zip › The raw date of WB - 上交/Figure 1 Differential expression of HSP90B1 in HNSC cell lines1-3.tif]

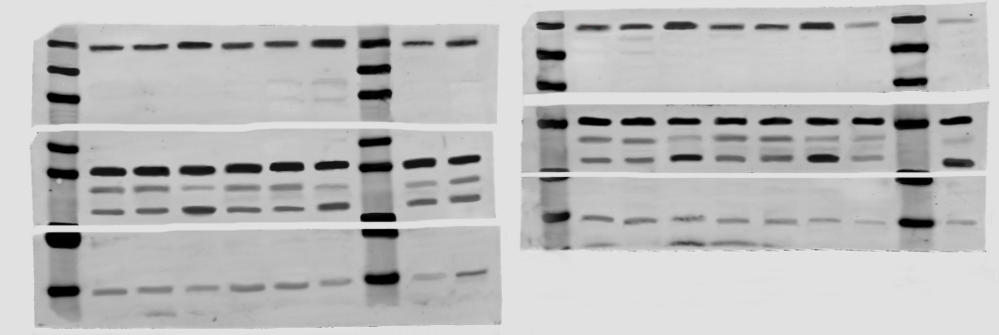

Supplement: Supplemental Information 1 — Repeated 3 times. [file peerj-12-17028-s001.zip › The raw date of WB - 上交/Figure 2 Apoptosis OE 2 20230518.tif]

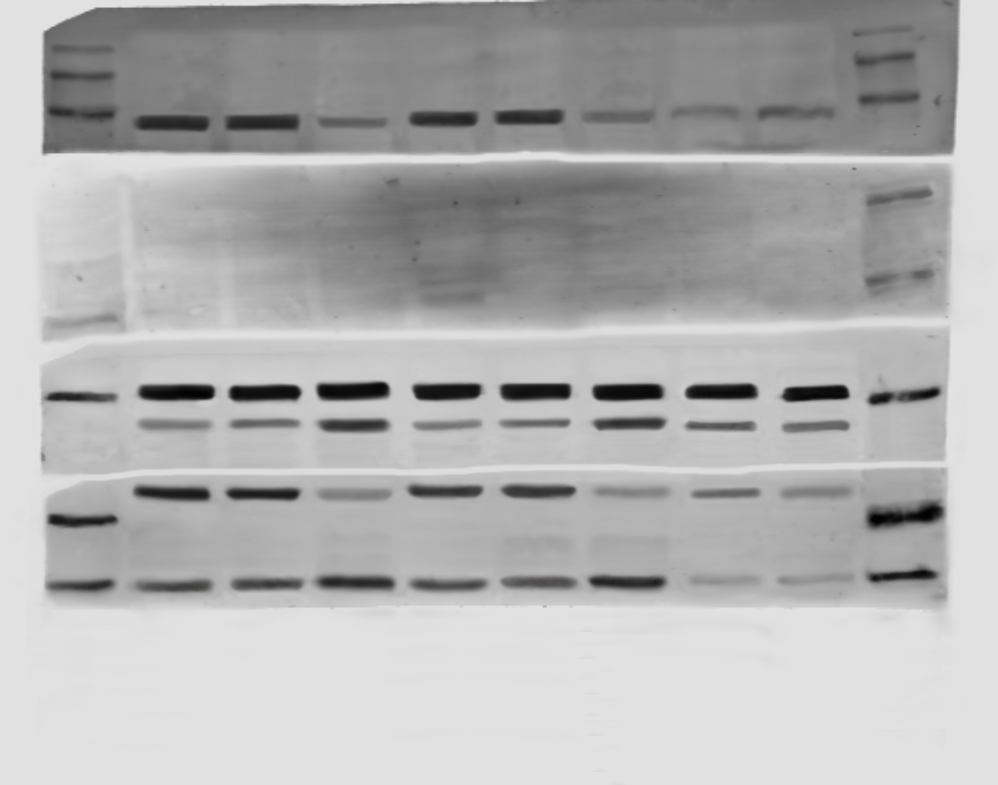

Supplement: Supplemental Information 1 — Repeated 3 times. [file peerj-12-17028-s001.zip › The raw date of WB - 上交/Figure 2 Apoptosis si 1.tif]

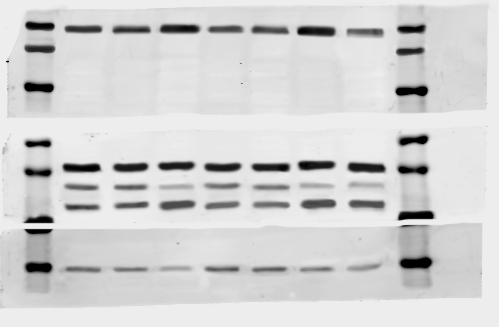

Supplement: Supplemental Information 1 — Repeated 3 times. [file peerj-12-17028-s001.zip › The raw date of WB - 上交/Figure 2 Apoptosis OE 1 20230518.tif]

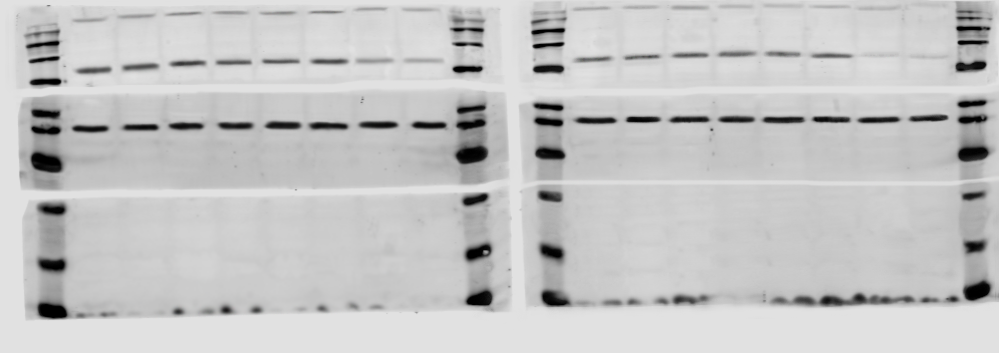

Supplement: Supplemental Information 1 — Repeated 3 times. [file peerj-12-17028-s001.zip › The raw date of WB - 上交/Figure 4 AKT mTOR pathway si 1-2.tif]

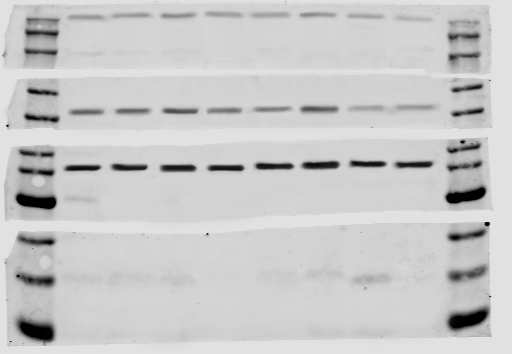

Supplement: Supplemental Information 1 — Repeated 3 times. [file peerj-12-17028-s001.zip › The raw date of WB - 上交/Figure 4 AKT mTOR pathway si 3.tif]

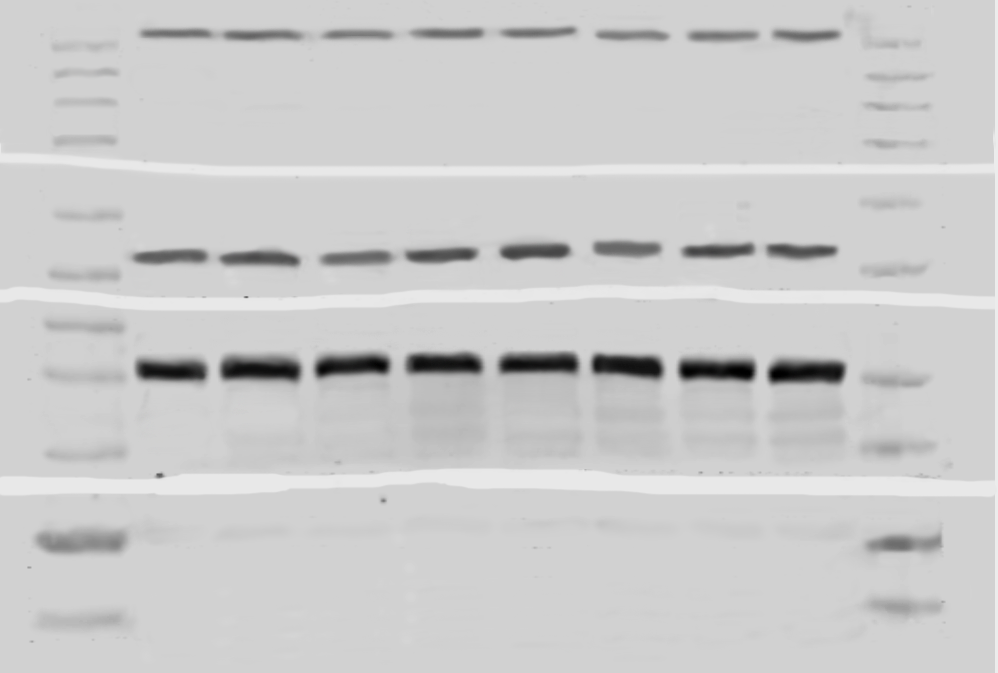

Supplement: Supplemental Information 1 — Repeated 3 times. [file peerj-12-17028-s001.zip › The raw date of WB - 上交/Figure 4 AKT mTOR OE 1.tif]

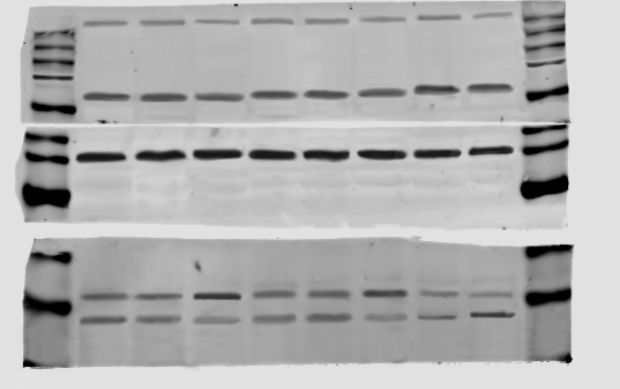

Supplement: Supplemental Information 1 — Repeated 3 times. [file peerj-12-17028-s001.zip › The raw date of WB - 上交/Figure 4 AKT mTOR OE 2.tif]

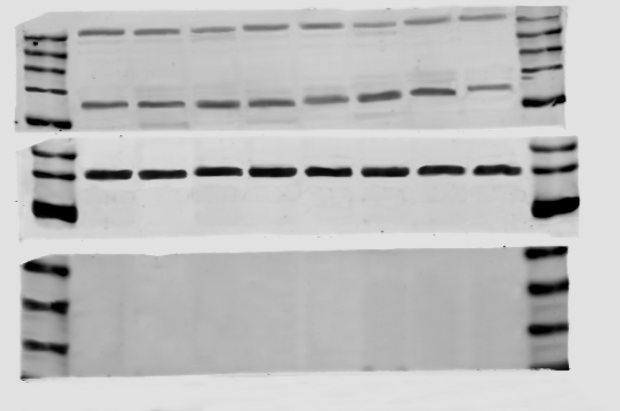

Supplement: Supplemental Information 1 — Repeated 3 times. [file peerj-12-17028-s001.zip › The raw date of WB - 上交/Figure 4 AKT mTOR OE 3.tif]

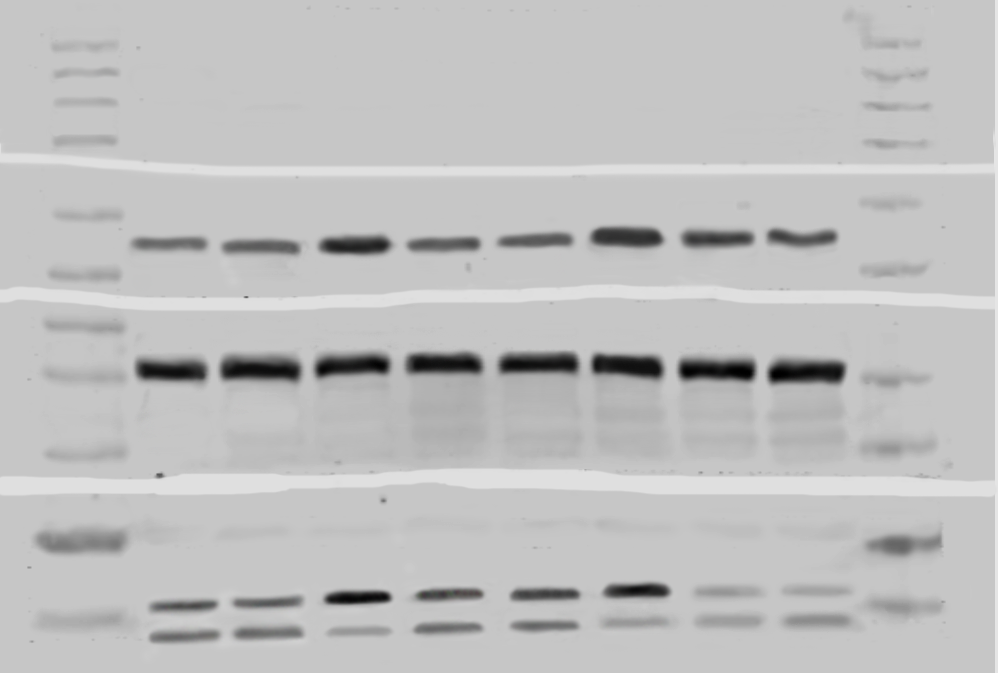

Supplement: Supplemental Information 1 — Repeated 3 times. [file peerj-12-17028-s001.zip › The raw date of WB - 上交/Figure 4 Autophagy OE 1.tif]

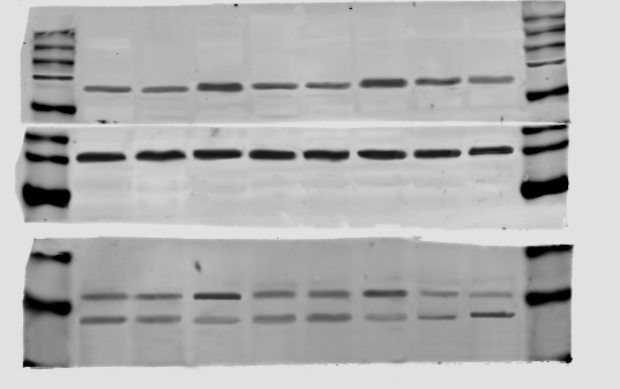

Supplement: Supplemental Information 1 — Repeated 3 times. [file peerj-12-17028-s001.zip › The raw date of WB - 上交/Figure 4 Autophagy OE 2.tif]

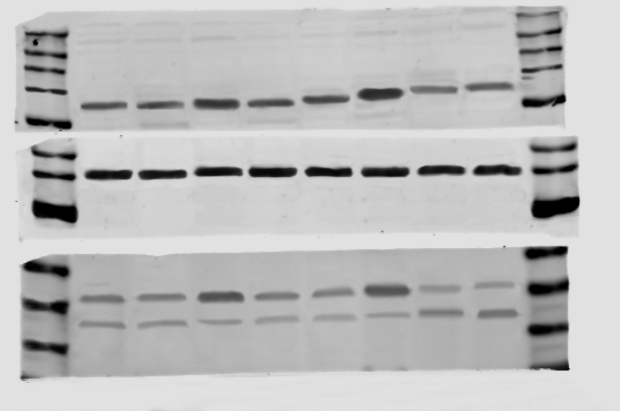

Supplement: Supplemental Information 1 — Repeated 3 times. [file peerj-12-17028-s001.zip › The raw date of WB - 上交/Figure 4 Autophagy OE 3.tif]

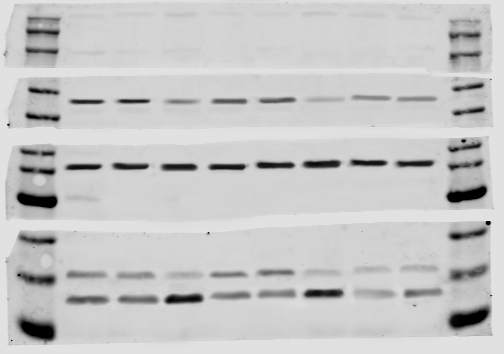

Supplement: Supplemental Information 1 — Repeated 3 times. [file peerj-12-17028-s001.zip › The raw date of WB - 上交/Figure 4 Autophagy si 3.tif]

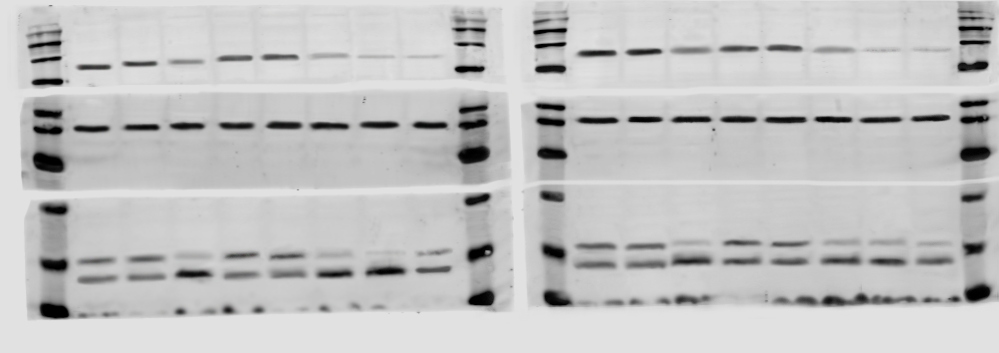

Supplement: Supplemental Information 1 — Repeated 3 times. [file peerj-12-17028-s001.zip › The raw date of WB - 上交/Figure 4 Autophagy si 1-2.tif]

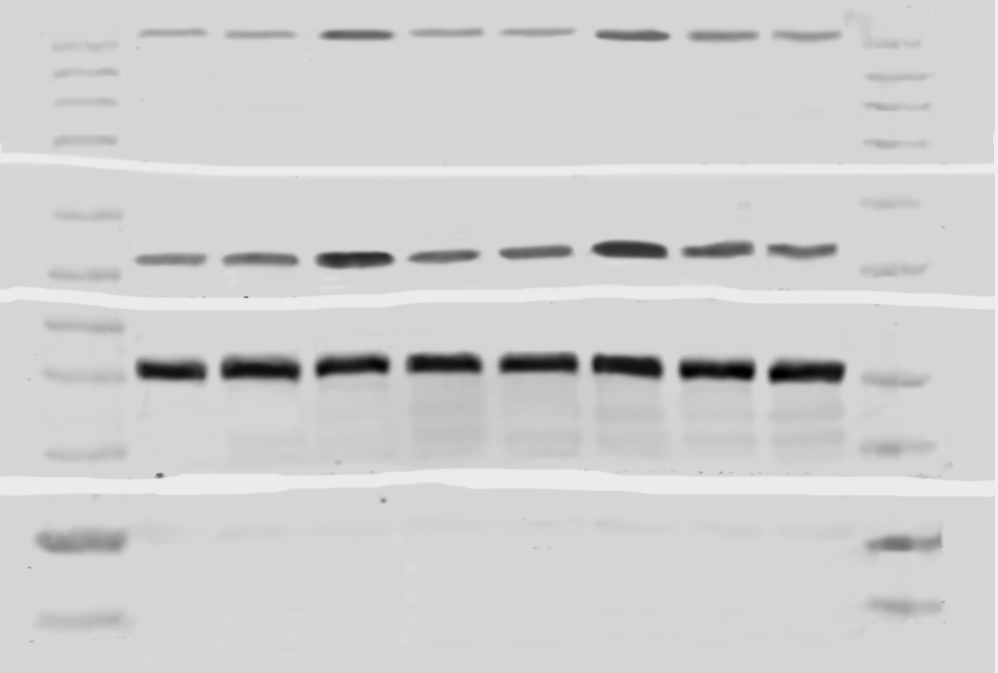

Supplement: Supplemental Information 1 — Repeated 3 times. [file peerj-12-17028-s001.zip › The raw date of WB - 上交/Figure 4 p-AKT mTOR OE 1 -.tif]

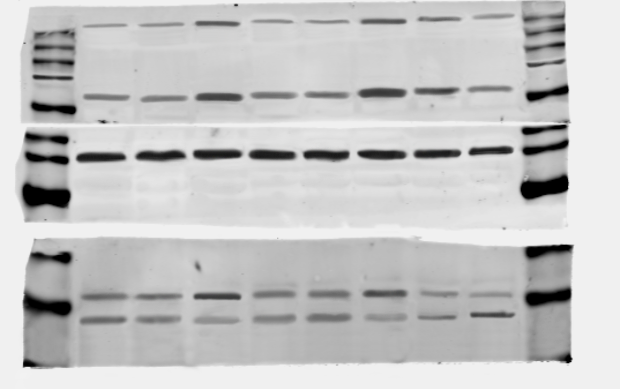

Supplement: Supplemental Information 1 — Repeated 3 times. [file peerj-12-17028-s001.zip › The raw date of WB - 上交/Figure 4 p-AKT mTOR OE 2 -.tif]

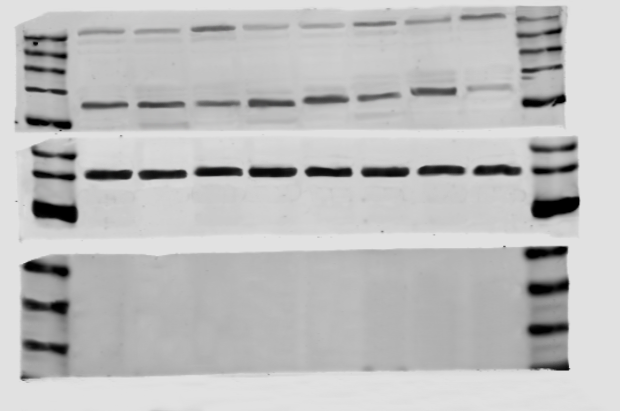

Supplement: Supplemental Information 1 — Repeated 3 times. [file peerj-12-17028-s001.zip › The raw date of WB - 上交/Figure 4 p-AKT mTOR OE 3.tif]

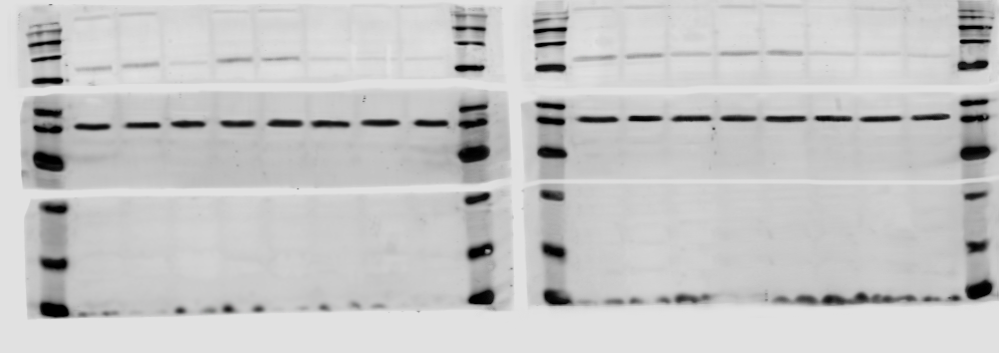

Supplement: Supplemental Information 1 — Repeated 3 times. [file peerj-12-17028-s001.zip › The raw date of WB - 上交/Figure 4 phosphorylation-AKT mTOR pathway si 1-2.tif]

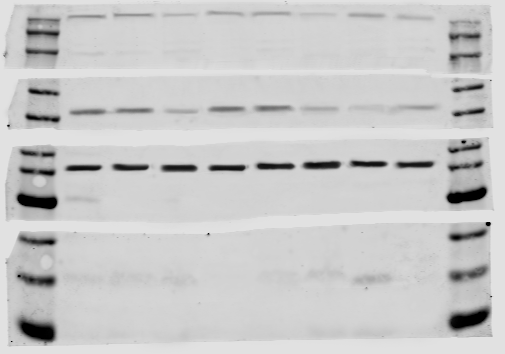

Supplement: Supplemental Information 1 — Repeated 3 times. [file peerj-12-17028-s001.zip › The raw date of WB - 上交/Figure 4 phosphorylation-AKT mTOR pathway 3.tif]

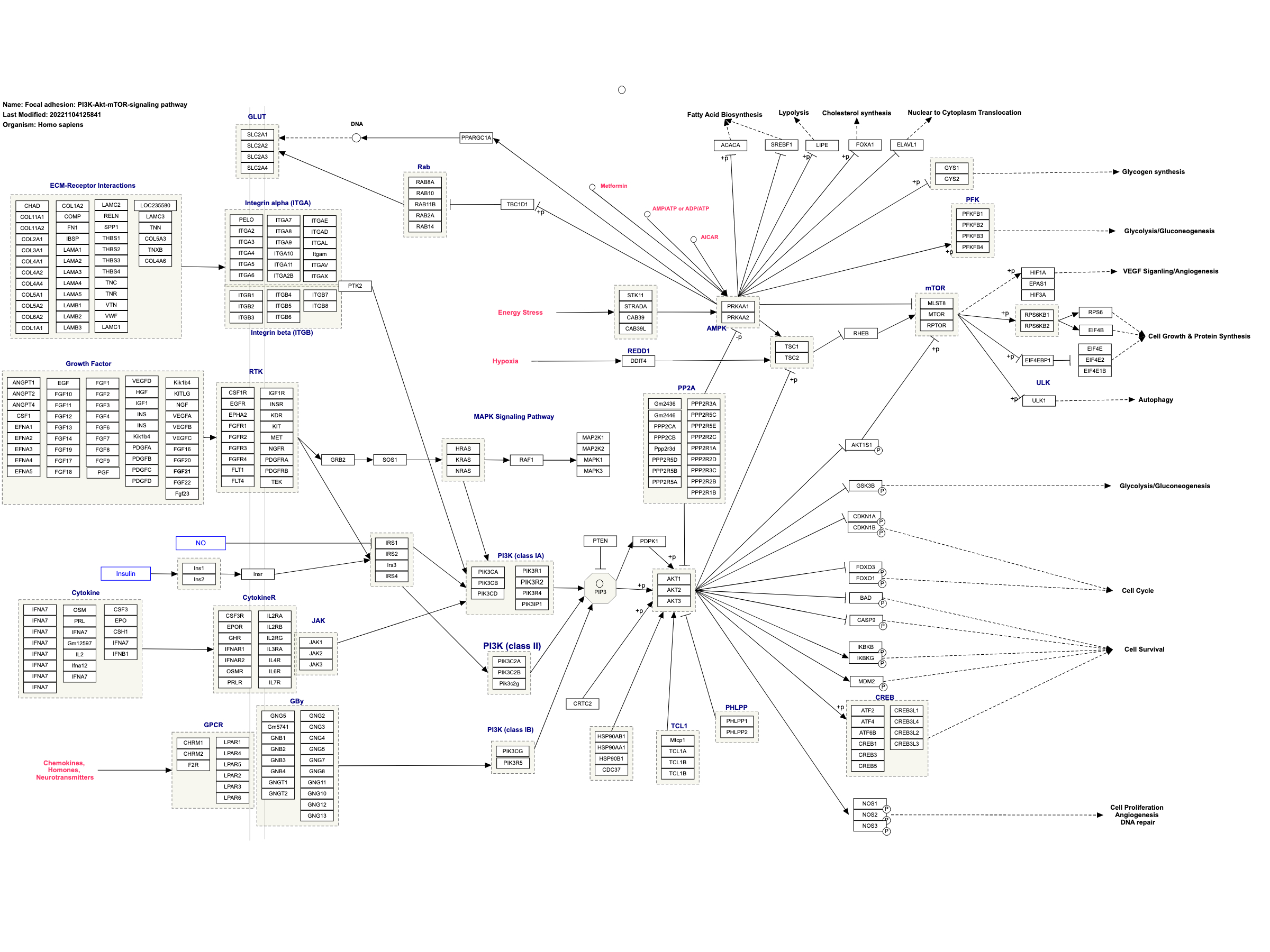

Supplement: Supplemental Information 3 — Wikipathway shows that HSP90B1 is involved in the PI3K/AKT/mTOR signaling pathway. [file peerj-12-17028-s003.jpg]
